# Prevalence and Overlap of Malnutrition, Sarcopenia and Related Nutritional Syndromes in a Large Haemodialysis Population

**DOI:** 10.1002/jcsm.70386

**Published:** 2026-09-28

**Authors:** Hanaa Noor, Subhashini Ganesan, Flavia Martinez Cantarutti, Ali Alharbi, Imed Helal, Joanne Reid, Adrian Slee

**Affiliations:** ^1^ Diaverum Riyadh Saudi Arabia; ^2^ Insights Research Organization and Solutions (IROS) Abu Dhabi UAE; ^3^ School of Midwifery and Nursing Queen's University Belfast Belfast UK; ^4^ Division of Medicine, Faculty of Medical Sciences University College London (UCL) London UK

**Keywords:** cachexia, end‐stage kidney disease, haemodialysis, malnutrition, sarcopenia, sarcopenic obesity

## Abstract

**Background:**

Chronic kidney disease is a major global health burden linked to malnutrition, sarcopenia and related nutritional syndromes including protein‐energy wasting (PEW)/cachexia and sarcopenic obesity (SO). Data from end‐stage kidney disease (ESKD) populations in Saudi Arabia and the wider Middle East are scarce. This study assessed the prevalence and factors associated with malnutrition and sarcopenia risk in a large haemodialysis (HD) cohort and the co‐occurrence of PEW/cachexia risk and SO.

**Methods:**

Multicentre, cross‐sectional study in 40 dialysis clinics across Saudi Arabia (June–August 2024). Adults on maintenance HD ≥ 90 days were screened using the malnutrition–inflammation score (MIS; high risk > 5), SARC‐F (sarcopenia risk ≥ 4) high risk, calf circumference (CC; cut‐offs ≤ 34‐cm males, ≤ 33‐cm females), CC adjusted for body mass index (BMI) and oedema (Adj. CC) and SARC‐CalF (sarcopenia risk ≥ 11). SO was defined as sarcopenia risk by SARC‐CalF (Adj. CC) plus BMI > 30 kg/m^2^. Routine blood chemistry (e.g., total iron‐binding capacity [TIBC] and albumin) and screening tools were collected for physical function (Karnofsky), falls risk (Morse fall) and comorbidity (Charlson index). Univariate and multivariate logistic regression (adjusted for age, sex, diabetes, dialysis vintage and Kt/V) identified factors associated with each outcome.

**Results:**

Of 3722 HD patients (56.8% male; age 53.6 ± 15.8 years; BMI 26.8 ± 6.4 kg/m^2^), 48% had diabetes and 35% had high/very high Charlson comorbidity index. High nutritional risk (MIS > 5) was found in 44.5% (*n* = 1657) and sarcopenia risk (SARC‐CalF [Adj. CC]) in 53.1% (*n* = 1978); 31.6% (*n* = 1175) had combined malnutrition and sarcopenia (M + S) and 7.7% (*n* = 285) had SO. Versus the no‐risk group, M + S patients had lower albumin, TIBC, Karnofsky score and higher comorbidity index and Morse fall risk score (all *p* < 0.001). Independent factors associated with high nutritional risk included lower BMI, higher Charlson index, longer dialysis vintage, lower serum creatinine and higher triglycerides (all *p* < 0.05). Shared independent factors for sarcopenia risk were older age, higher comorbidity index, longer dialysis duration, lower creatinine and higher triglycerides (all *p* < 0.05).

**Conclusions:**

This is the first large‐scale study to fully characterise a Saudi and Middle Eastern HD cohort. There was a very high risk of malnutrition and sarcopenia. Furthermore, a high number had both conditions and overlapping features of PEW/cachexia, which included high comorbidity and disability risk. Findings support routine, multitool nutritional and muscle screening in HD care, including younger patients.

## 1 Introduction

Approximately 10% of the global population suffers from chronic kidney disease (CKD) [1]. In the Kingdom of Saudi Arabia (KSA), CKD is increasingly recognised as a significant public health concern, with prevalence estimates of 4.76% among adults [2]. The burden is particularly notable among older adults and those with diabetes or hypertension, reflecting global trends in CKD epidemiology [2]. By the end of 2020, there were more than 20 000 patients with end‐stage kidney disease (ESKD) receiving dialysis in KSA [3].

CKD is associated with a range of deleterious nutritional syndromes in patients, such as malnutrition, cachexia, sarcopenia and frailty, along with subcategories including ‘protein‐energy wasting’ (PEW) and ‘sarcopenic obesity’ (SO) [4, 5, 6]. Higher rates of these conditions tend to be observed in the ESKD dialysis population due to a complex interplay between the disease itself (e.g., uremic toxins, acidosis and inflammation), treatment modalities (e.g., dialysis and subsequent nutrient losses of amino acids), physical inactivity and sedentary behaviour and comorbidities (e.g., diabetes and cardiovascular disease). These conditions worsen clinical outcomes and reduce quality of life [4, 7, 8, 9]. Recent literature proposes that PEW and cachexia exist on a continuum, with the overarching term ‘kidney disease cachexia’ encompassing PEW progressing to cachexia [10].

Screening and assessment for these nutrition‐related conditions in ESKD dialysis patients has become a clinical priority due to its impact on outcomes. The latest Kidney Disease Outcomes Quality Initiative (KDOQI) 2020 nutrition in CKD guidelines highlights the importance of routine nutritional screening for dialysis patients using validated tools such as the subjective global assessment (SGA) or the malnutrition–inflammation score (MIS)/KALANTAR score, that incorporate multiple markers rather than single measures (e.g., bodyweight) [11]. The MIS/KALANTAR tool has been validated in CKD stage 5D HD patients; the scoring system is derived from the SGA, alongside comorbidities, body mass index (BMI) and serum albumin, total iron‐binding capacity (TIBC) as inflammatory markers [12]. It was recently utilised in a large HD population (2975 patients) in Diaverum dialysis clinics in Portugal [13]. Both the MIS and the 7‐point SGA have recently been compared with the global leadership initiative for malnutrition (GLIM) criteria in haemodialysis (HD) patients (cohorts from Italy and Brazil), concluding that the 7‐point SGA and MIS are superior [14]. Data on malnutrition risk in Saudi HD patients remain limited to small single‐ or two‐centre studies using SGA, anthropometry and plasma albumin. The most recent study by Azzeh et al. reported 54.5% of patients with some level of malnutrition [15].

Sarcopenia is a progressive muscle disease associated with ageing and chronic conditions such as CKD, defined by three features: loss of muscle strength, skeletal muscle mass (MM) and physical performance [16]. It is associated with adverse outcomes, including increased risk of frailty, falls, fractures, physical disability and mortality, as well as reduced quality of life [16]. Previous studies have reported higher levels of sarcopenia in HD patients, which may vary depending on the criteria used [17, 18, 19]. Sarcopenia data in the Saudi general and HD populations remain scarce and require urgent investigation. Ongoing efforts aim to establish population‐specific sarcopenia cut‐off points that accurately reflect the genetic and phenotypic make‐up of the Saudi population [20].

Sarcopenia screening in HD patients can be performed using the simple SARC‐F five‐item questionnaire (strength, assistance with walking, rising from chair, climbing stairs and falls) combined with calf circumference (CC) measurement as a proxy for MM; these tools are rapid, non‐invasive, cost‐effective and portable [21, 22]. The SARC‐CalF tool, developed by Barbosa‐Silva et al., combines the SARC‐F and CC on a 0–20‐point scale. The SARC‐F has recently been shown to predict sarcopenia in a Chinese HD cohort [23], and both the SARC‐F and SARC‐CalF predict sarcopenia‐related traits (e.g., low hand grip strength, five‐times sit‐to‐stand test and gait speed) in HD patients [24]. In addition, Borges et al. demonstrated that the SARC‐CalF is a good predictor of mortality in Brazilian HD patients [25].

Establishing national prevalence data for malnutrition, sarcopenia and related nutritional syndromes in HD patients remains a critical gap in Saudi Arabia. Diaverum Saudi Arabia is the largest independent kidney care provider in the Kingdom, serving approximately 25% of the dialysis population and offering a large, multicentre platform to implement and evaluate routine screening and intervention models tailored to local needs [26]. This study therefore aimed to (i) characterise a large Saudi HD cohort; (ii) determine the prevalence of malnutrition and sarcopenia using the MIS, CC, adjusted CC, SARC‐F and SARC‐CalF (Adj. CC); and (iii) estimate the co‐occurrence of related nutritional syndromes, including PEW/cachexia risk and SO. A secondary objective was to identify factors associated with malnutrition and sarcopenia risk in this population.

## 2 Methods

### 2.1 Study Design

This study is part of a larger initiative called ‘Project Muscle’, which aims to evaluate and establish practical screening and intervention models for malnutrition and sarcopenia in dialysis care. Specifically, this paper reports data from a multicentre, cross‐sectional study conducted across 40 Diaverum dialysis clinics in 33 cities across KSA, integrating multiple validated tools for the screening of malnutrition and sarcopenia in alignment with international guidelines (KDOQI Clinical Practice Guideline for Nutrition in CKD: 2020 Update and the Revised European Consensus on Definition and Diagnosis of Sarcopenia—EWGSOP2) [11, 16]. Baseline data were collected between June and August 2024. Ethical approval was granted by the King Fahad Medical City Institutional Review Board (approval numbers 21‐519E and 24‐715E), and all participants provided written informed consent.

### 2.2 Study Population and Recruitment

A total of 4330 adults undergoing maintenance HD were screened. Eligible participants were identified by the clinical dietitian and nephrologist team at each dialysis unit. Patients were approached during their regular dialysis sessions, and written informed consent was obtained by trained healthcare professionals. Inclusion criteria were age ≥ 18 years, receiving thrice‐weekly in‐centre HD for at least 90 days and able to provide written informed consent. Exclusion criteria were pregnancy, severe physical or cognitive impairment (e.g., nonambulatory patients or those with documented dementia). After applying these criteria, 3722 patients were included in the final analysis (Figure 1).

**FIGURE 1:**
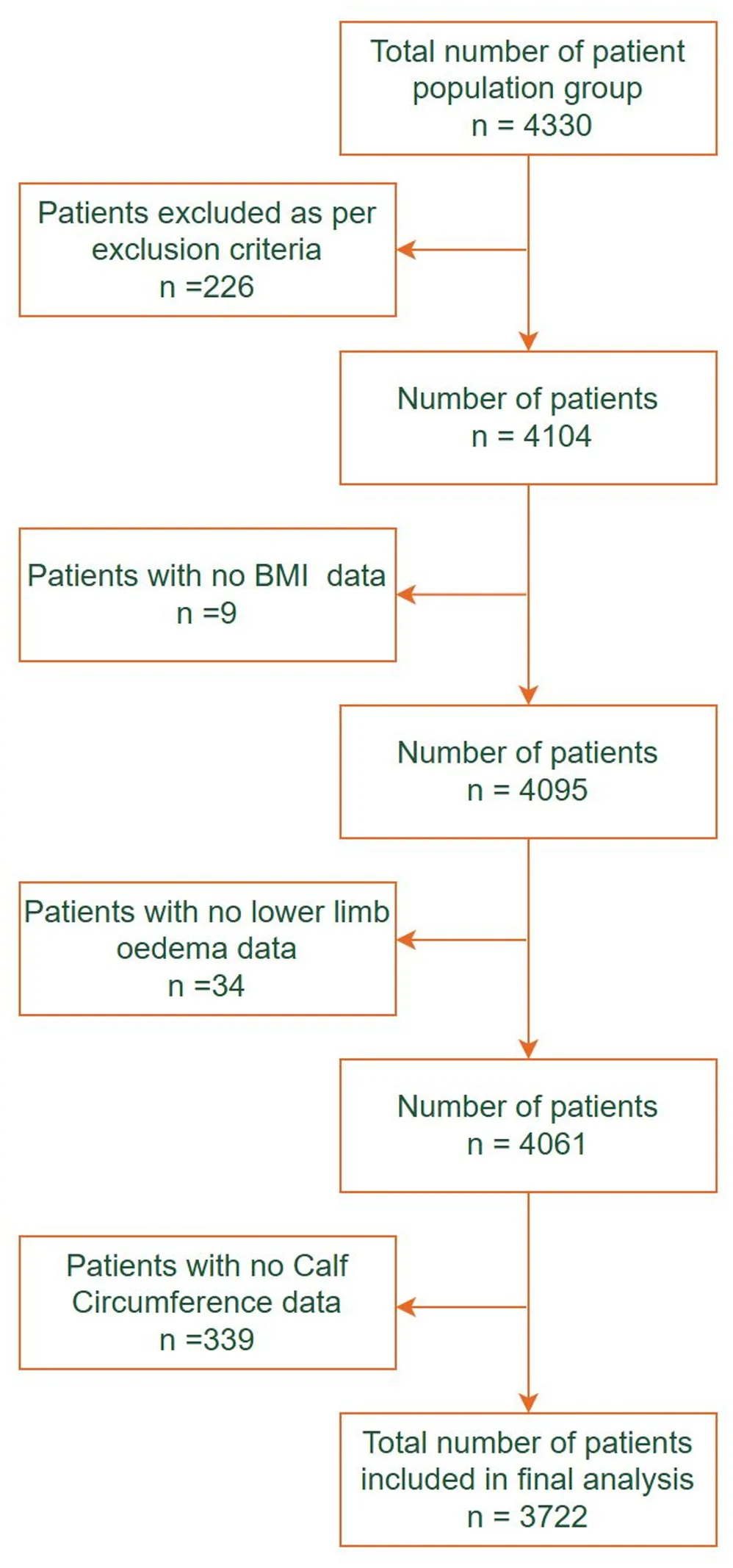
Study flow diagram: patient screening and inclusion for analysis.

### 2.3 Staff Training, Standardisation and Data Collection

Data were collected by 55 clinical dietitians across the 40 sites, following a written standardised operating procedure (SOP) and site‐specific reference materials. All dietitians completed a structured training programme comprising two components: a 3‐day centralised online training on renal nutrition, malnutrition and sarcopenia in CKD, delivered by an externally recognised expert renal dietitian and preceded by pre‐recorded preparatory content. These were followed by a 3‐day study‐specific training on HD measurement protocols, tool administration and data entry for all dietitians, treating physicians and nursing staff led by the lead author and a consultant nephrologist. All sessions were recorded for reference.

To standardise anthropometric assessment, identical stadiometers and weighing scales (same manufacturer and model) were installed across all 40 clinics, and all patient heights and body weights remeasured; dietitian assessments were cross‐checked by the treating physician at each site. Prior to main‐study enrolment, the screening protocol and measurement procedures were piloted in a smaller cohort of HD patients (*n* = 392) across three sites, during which malnutrition and sarcopenia indicators (e.g., bioelectrical impedance assessment and anthropometry) were compared with confirm clinical feasibility, patients' acceptability and indication of accuracy and reliability of data. A centralised electronic data‐collection platform was used, with weekly central review by the lead author and validation reports returned to each site throughout the 3‐month study period. These combined measures were used to minimise measurement variability across sites.

### 2.4 Medical History, Dialysis Information and Laboratory Values

Routinely collected clinical data were extracted for each patient, comprising demographic and medical history variables (age, sex, diabetes, Charlson comorbidity index [CCI] [scoring based upon 19 clinical conditions, with CCI scores of 1–2; moderate, with CCI scores of 3–4; and severe, with CCI scores ≥ 5] [27], Karnofsky scale for functional status [0%–100% scale, where 100% indicates complete independence and 0% indicates death] [28] and Morse fall risk score [0–125 scale based upon six components, > 51 indicates high risk of falls] [29]); dialysis‐related variables (dialysis vintage, modality, primary vascular access, interdialytic weight gain [IDWG], weekly treatment time, dialysis adequacy [Kt/V] and urea reduction ratio [URR]); and laboratory values (predialysis serum creatinine, urea, calcium, phosphorus, calcium–phosphorus product, potassium, intact parathyroid hormone [PTH], normalised protein catabolic rate [nPCR], haemoglobin, albumin, TIBC, LDL and HDL cholesterol, triglycerides, 25‐OH vitamin D3 and weekly erythropoietin [EPO] dose).

### 2.5 Anthropometric Measurements

Anthropometric indices include height, weight and BMI, calculated as body in kilogram divided by height squared in metre (kg/m^2^), and CC, measured in cm using a non‐elastic tape measure (see below for more details).

### 2.6 Malnutrition/Nutritional‐Risk Status

Malnutrition status was identified using the MIS. The MIS was selected as the primary malnutrition screening tool because it is validated in CKD stage 5D HD patients [11]; provides a quantitative composite of SGA‐derived items, comorbidity, anthropometrics and inflammatory biomarkers; has been successfully applied at scale in a recent comparative HD study [13]; and has been shown to outperform GLIM criteria in a recent comparative HD study [14]. Based on existing data, we defined as absence of nutritional risk by MIS ≤ 5 and a MIS > 5 with high nutritional risk noting that MIS score of > 5 had a significant risk of 1‐year mortality [30].

### 2.7 Sarcopenia Risk Status

Sarcopenia risk was screened using the SARC‐F 5‐question screening form which asks about strength, assistance with walking, rising from a chair, climbing stairs and falls. SARC‐F is scored 0–10 (2 points per question) with a score of ≥ 4 indicating possible sarcopenia presence [21]. CC (see below) was then combined with SARC‐F to derive two composite tools: SARC‐CalF (SARC‐F + CC) and SARC‐CalF (Adj. CC) (SARC‐F + BMI–oedema–adjusted CC) [22]. For both composites, a total score of 0–10 indicates low sarcopenia risk and 11–20 indicates possible sarcopenia.

### 2.8 Calf Measurements

CC was measured predialysis either standing or seated with foot resting on the floor (when unable to stand with or without support or if wheel chair bound) as a marker for MM, with cut‐off points utilised based upon analysis of US NHANES data indicating low MM/moderate sarcopenia, that is, ≤ 34 cm for males and ≤ 33 cm for females [31], noting the current lack of KSA population–specific references. In addition, CC was adjusted for BMI and oedema based upon previous estimates from research.

*Adjusted CC in case of excess weight* [32]: BMI 25–29.9 kg/m^2^: subtract 3 cm; BMI 30–39.9 kg/m^2^: subtract 7 cm; BMI ≥ 40 kg/m^2^: subtract 12 cm.

*Adjusted CC in case of oedema* [33]: Males: subtract 2 cm and females: subtract 1.6 cm.

### 2.9 Screening and Diagnosis Pathway

Figure 2 shows the overall risk classification for malnutrition and sarcopenia including screening and diagnosis, along with specific nutritional syndromes, such as SO.

**FIGURE 2:**
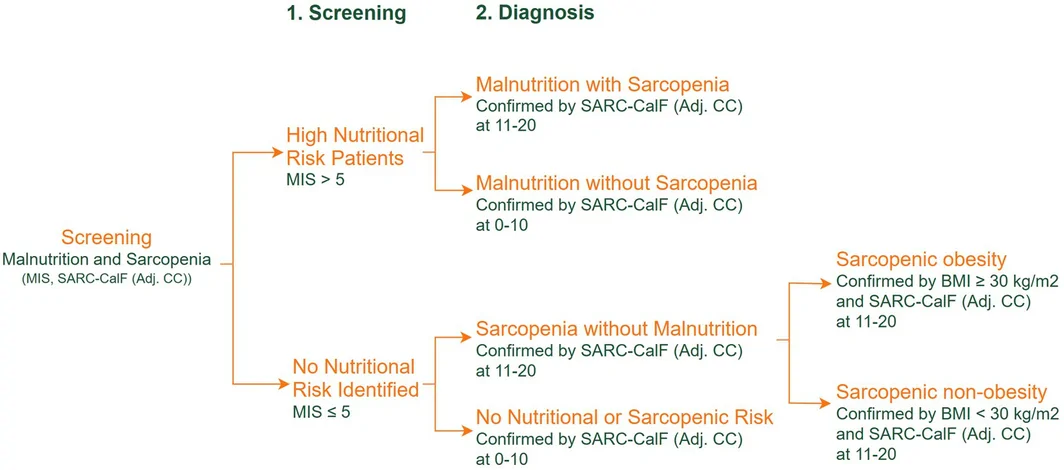
Algorithm diagram for malnutrition and sarcopenia screening. BMI: body mass index, CC: calf circumference, MIS: malnutrition–inflammation score, SARC‐F: strength, assistance walking, rising from chair, climbing stairs and falls questionnaire.

### 2.10 Statistical Analysis

Statistical analysis was performed using IBM SPSS Statistics for Windows, Version 30.0 (IBM Corp., Armonk, NY, USA) and R Statistical Software Version 4.5.0.

The distribution of these groups among the study population is presented as frequencies and percentages (*n* [%]). Continuous data are presented as means ± standard deviations and were analysed using Student's *t*‐test. Categorical data are shown as *n* (%) and were analysed using the chi‐square test.

Univariate binary logistic regression was used to analyse association of factors (demographic and clinical factors) with the development of nutritional risk and sarcopenia among dialysis patients. Based on clinical expertise and previous research, relevant factors that could impact the development of nutritional risk and sarcopenia among dialysis patients were decided for univariate analysis a priori. Factors such as albumin and TIBC were not included as individual variables in the analysis as they were used to derive the composite MIS score. Including both would introduce redundancy and collinearity, potentially biasing estimates; therefore, only the composite score was retained in the analysis.

A multivariate logistic regression model was used to assess the independent association of variables that were statistically significant in the univariate analysis (*p* < 0.05) and were adjusted for age, gender, diabetic status, dialysis vintage and Kt/V. Two‐sided *p* values < 0.05 were considered statistically significant and unadjusted and adjusted odds ratios (ORs) are reported for univariate and multivariate analysis, respectively, with corresponding 95% confidence intervals (CIs). Multiple comparisons were adjusted using the Benjamini–Hochberg false discovery rate (FDR) method at a significance level of 5%, and corrected *p* values are provided.

## 3 Results

### 3.1 General Characteristics of Study Population

A total of 3722 HD patients who were all KSA nationals were included in this study analysis, of which 2114 (56.8%) were male, and the average age of the participants was 53.6 years. The majority of the study cohort had an autologous arteriovenous (AV) fistula (80.9%) as their primary vascular access and underwent high‐flux HD (63.9%). Thirty‐five per cent of the cohort were classified as being either high (23.6%) or very high (11.4%) in their comorbidity index according to the CCI, and nearly half of the population (48%) had history of diabetes. In the study cohort, we found risk of fall (low to high risk of fall according to Morse fall score), average BMI and nutritional risk (based on mean CC and scoring for SARC‐F, SARC‐Calf and SARC‐CalF [Adj. CC]) were significantly higher in females compared with males (*p* < 0.001). No significant difference between gender was observed for age, CCI and adjusted CC and MIS score. Additionally, Karnofsky functionality score is significantly higher in males compared with females along with distribution of factors such as HDF and autologous AV fistula as primary vascular access were significantly higher in males compared with female dialysis patients (*p* < 0.001). The general characteristics of the study population are presented in Table 1.

**TABLE 1:** General characteristics of the participant population.

Demographic characteristics	Total *N* of patients	Female	Male	*p*
	3722 (100)	1608 (43.2)	2114 (56.8)	
Age (years), mean ± SD	53.6 ± 15.8	54.7 ± 15.9	52.7 ± 15.8	0.530
Age group, *n* (%)				
< 18 years	15 (0.4)	8 (0.5)	7 (0.3)	
18–39 years	758 (20.4)	291 (18.1)	467 (22.1)	
40–64 years	1980 (53.2)	841 (52.3)	1139 (53.9)	
≥ 65 years	969 (26)	468 (29.1)	501 (23.7)	
Body mass index (kg/m^2^)	26.8 ± 6.4	27.2 ± 7	26.5 ± 5.9	< 0.001*
Underweight (< 18.5)	262 (7)	135 (8.4)	127 (6)	
Healthy weight (18.5–24.9)	1321 (35.5)	534 (33.2)	787 (37.2)	
Overweight (25.0–29.9)	1138 (30.6)	440 (27.4)	698 (33)	
Obese (≥ 30.0)	1001 (26.9)	499 (31)	502 (23.7)	
Charlson comorbidity index	5 ± 2.2	5 ± 2.2	5 ± 2.2	0.439
Low (1–3)	1350 (36.3)	562 (35)	788 (37.3)	
Moderate (4–5)	1069 (28.7)	453 (28.2)	616 (29.1)	
High (6–7)	879 (23.6)	398 (24.8)	481 (22.8)	
Very high (≥ 8)	424 (11.4)	195 (12.1)	229 (10.8)	
Karnofsky index, mean ± SD	83.6 ± 15.8	80 ± 16.8	86.3 ± 14.3	< 0.001*
Morse Fall Scale score, *n* (%)				
No risk	1569 (42.2)	609 (37.9)	960 (45.4)	
Low to moderate risk	1905 (51.2)	864 (53.7)	1041 (49.2)	< 0.001*
High risk	247 (6.6)	135 (8.4)	112 (5.3)	< 0.001*
Not available	1 (0.03)		1 (0.05)	
History of diabetes				0.716
No	1936 (52)	842 (52.4)	1094 (51.8)	
Yes	1786 (48)	766 (47.6)	1020 (48.2)	
HD modality type				< 0.001*
HDF	1342 (36.1)	481 (29.9)	861 (40.7)	
High‐flux haemodialysis	2380 (63.9)	1127 (70.1)	1253 (59.3)	
Months on dialysis, mean ± SD	71.1 ± 62.1	74.7 ± 63.2	68.4 ± 61	0.066
Primary vascular access				< 0.001*
Autologous AV fistula	3010 (80.9)	1247 (77.5)	1763 (83.4)	
AV graft	147 (3.9)	71 (4.4)	76 (3.6)	
Tunnelled catheter	565 (15.2)	290 (18)	275 (13)	
Sarcopenia and malnutrition parameters				
Calf circumference (CC in cm)	32.5 ± 5.2	32.7 ± 5.5	32.4 ± 5	< 0.001*
Adjusted calf circumference (Adj. CC in cm)	29.4 ± 4.1	29.3 ± 4.1	29.5 ± 4	0.37
SARC‐F	2.2 ± 2.6	2.8 ± 2.8	1.7 ± 2.4	< 0.001*
SARC‐CalF	8.6 ± 5.6	8.6 ± 5.8	8.6 ± 5.5	< 0.001*
SARC‐CalF (Adj. CC)	11 ± 4.3	11.3 ± 4.7	10.8 ± 4	< 0.001*
MIS score	5.8 ± 3.1	6.2 ± 3.2	5.4 ± 3	0.078

* *p* < 0.001.

### 3.2 Prevalence of Malnutrition and Sarcopenia

Among the 3722 dialysis patients, 1175 patients (31.6%) were found to have both malnutrition and sarcopenia and 482 patients (13%) exhibited malnutrition without sarcopenia features. Notably, 1262 patients (33.9%) did not present with either malnutrition or sarcopenia according to the MIS and sarcopenic criteria. Furthermore, sarcopenia without malnutrition was identified in 803 patients (21.6%). This subgroup included 285 patients with SO (7.7%) and 518 (13.9%) with sarcopenic nonobesity (Figure 3).

**FIGURE 3:**
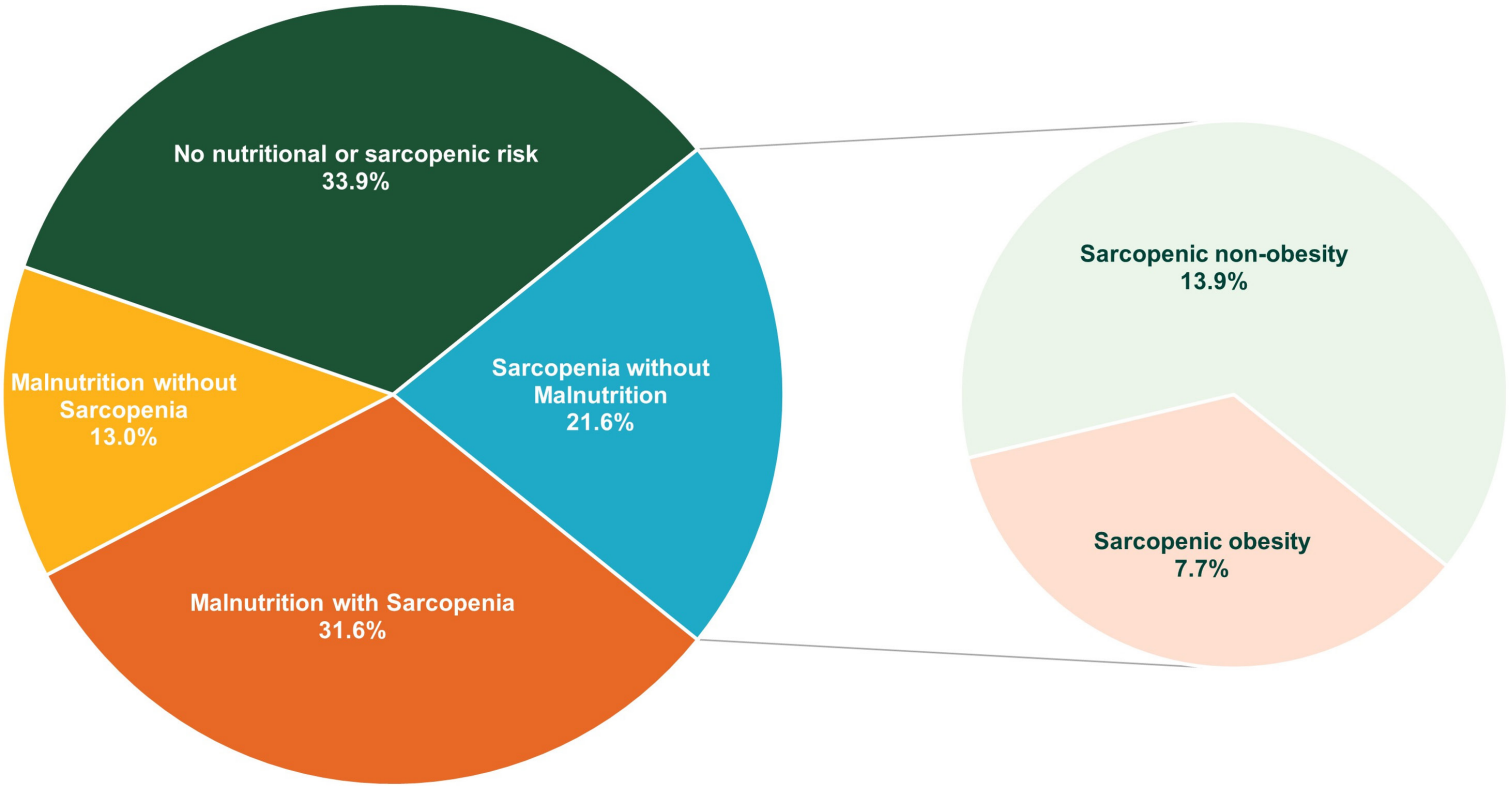
Prevalence of malnutrition and sarcopenia and subcategories.

### 3.3 Prevalence (%) of Nutritional Risk and Sarcopenia Based on Various Criteria

Specific categorisation based upon MIS, CC, adjusted CC, SARC‐F, SARC‐CalF and SARC‐CalF (Adj. CC) can be found in Figure 4, showing varying prevalence. The SARC‐CalF (Adj. CC) criteria identified highest number of patients with sarcopenia constituting 53%.

**FIGURE 4:**
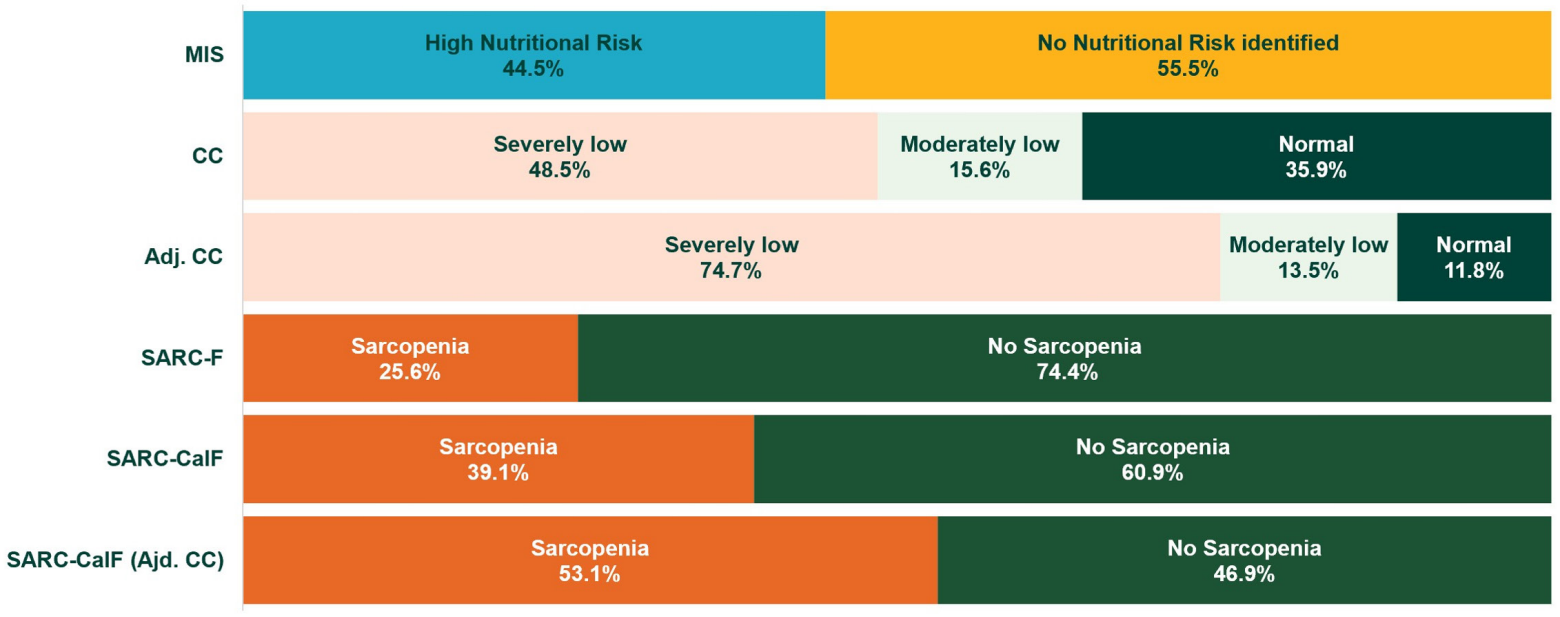
Prevalence (%) of nutritional risk and sarcopenia based on various criteria.

### 3.4 Malnutrition Analysis and Associated Risk Factors

In the study cohort, comprising 3722 HD patients, 1657 (44.5%) were identified to have high nutritional risk based on MIS criteria with scores exceeding five.

Univariate binary logistic regression was performed to understand the association of factors deemed clinically relevant for developing nutritional risk (Table 2). Several factors were found to be significantly associated with increased odds of developing high nutritional risk including advanced age, female gender, lower BMI, higher comorbidity index, history of diabetes, patients undergoing high‐flux HD, patients undergoing dialysis for longer period and lower average weekly treatment time. Biochemical parameters such as lower NPCR, lower creatinine levels, higher equilibrated Kt/V, higher urea reduction ratio, lower predialysis urea levels, lower phosphorus, lower calcium–phosphorus product, lower haemoglobin, higher HDL and higher triglycerides were associated with increased odds of developing higher nutritional risk.

**TABLE 2:** Regression analysis of factors associated with high nutritional risk among dialysis patients.

Total nossf patients, *n* (%)	High nutritional risk (MIS > 5)	No nutritional risk (MIS ≤ 5)	Univariate regression analysis		Multivariate regression analysis		
	1657 (44.5)	2065 (55.5)	Unadjusted OR (95% CI)	*p*	Adjusted OR (95% CI)	*p*	*p* (corrected for multiple testing using FDR)
Demographic variables							
Age, mean ± SD	57.7 ± 16.3	50.3 ± 14.7	1.031 (1.027–1.036)	< 0.001*	1.009 (1.001–1.016)	0.024*	0.054
Gender, *n* (%), (female vs. male)			1.465 (1.286–1.67)	< 0.001*	1.1 (0.924–1.311)	0.284	0.461
Female	802 (49.9)	806 (50.1)					
Male	855 (40.4)	1259 (59.6)					
Clinical variables							
Body mass index (kg/m^2^), mean ± SD	25.26 ± 6.33	28.01 ± 6.25	0.931 (0.92–0.941)	< 0.001*	0.923 (0.911–0.936)	< 0.001*	0.003*
Charlson comorbidity index, mean ± SD	5.27 ± 2.28	4.16 ± 1.97	1.275 (1.235–1.316)	< 0.001*	1.263 (1.176–1.357)	< 0.001*	0.003*
H/o diabetes, *n* (%), (yes vs. no)			1.501 (1.318–1.709)	< 0.001*	1.168 (0.931–1.466)	0.179	0.322
No	769 (39.7)	1167 (60.3)					
Yes	888 (49.7)	898 (50.3)					
Dialysis variables							
HD modality type, *n* (%), (HDF vs. high‐flux haemodialysis)			0.838 (0.732–0.959)	0.010*	0.849 (0.722–0.998)	0.047*	0.094
HDF	560 (41.7)	782 (58.3)					
High‐flux haemodialysis	1097 (46.1)	1283 (53.9)					
Months on dialysis, mean ± SD	79.0 ± 64.0	64.8 ± 59.7	1.004 (1.003–1.005)	< 0.001*	1.005 (1.003–1.006)	< 0.001*	0.003*
Primary vascular access type (at end of period), *n* (%), (autologous as reference category)							
Autologous AV fistula	1318 (43.8)	1692 (56.2)					
AV graft	71 (48.3)	76 (51.7)	1.199 (0.861–1.671)	0.282			
Tunnelled catheter	268 (47.4)	297 (52.6)	1.158 (0.967–1.387)	0.110			
IDWG (weight gain %), mean ± SD	2.18 ± 1.19	2.20 ± 1.13	0.992 (0.938–1.049)	0.774			
Weekly treatment time (average, last 4 weeks) (min), mean ± SD	717 ± 66.1	722 ± 60.7	0.999 (0.998–1)	0.011*	0.998 (0.997–0.999)	< 0.001*	0.003*
Labs, mean ± SD							
Normalised protein catabolic rate (g/kg/day)	0.96 ± 0.23	1.03 ± 0.24	0.274 (0.206–0.365)	< 0.001*	0.648 (0.07–6.031)	0.703	0.726
Creatinine‐S (mg/dL)	8.44 ± 2.4	9.85 ± 2.71	0.805 (0.783–0.827)	< 0.001*	0.888 (0.852–0.925)	< 0.001*	0.003*
Intact PTH (pg/mL)	397.95 ± 419.34	405.68 ± 352.45	1 (1–1)	0.541			
Equilibrated Kt/V	1.58 ± 0.24	1.53 ± 0.2	2.502 (1.849–3.386)	< 0.001*	1.358 (0.491–3.76)	0.556	0.715
Urea reduction ratio	0.78 ± 0.04	0.77 ± 0.04	126.43 (23.95–667.46)	< 0.001*	2.344 (0.02–272.879)	0.726	0.726
Predialysis urea (mmol/L)	16.9 ± 4.78	18.63 ± 4.96	0.928 (0.915–0.941)	< 0.001*	0.981 (0.884–1.089)	0.723	0.726
Calcium‐S (mg/dL)	9.05 ± 0.57	9.07 ± 0.56	0.935 (0.834–1.049)	0.252			
Phosphorus‐S, inorganic (mg/dL)	4.74 ± 0.95	4.97 ± 0.95	0.769 (0.716–0.826)	< 0.001*	1.111 (0.866–1.425)	0.408	0.565
Calcium phosphorus product (mg^2^/dL^2^)	43.01 ± 9.37	45.12 ± 9.03	0.975 (0.968–0.982)	< 0.001*	0.987 (0.962–1.012)	0.307	0.461
Haemoglobin‐B (g/dL)	10.80 ± 0.99	10.89 ± 0.91	0.907 (0.847–0.972)	0.005*	0.913 (0.844–0.988)	0.024*	0.054
Vitamin D3, 25‐hydroxy‐S (nmol/L)	71.17 ± 46.54	71.95 ± 48.13	1 (0.998–1.001)	0.618			
HDL cholesterol‐S (mg/dL)	44.02 ± 13.65	42.65 ± 13.22	1.008 (1.003–1.012)	0.002*	0.999 (0.993–1.004)	0.624	0.726
Triglycerides (mg/dL)	59.39 ± 80.31	53.68 ± 90.9	1.001 (1–1.002)	0.045*	1.002 (1.001–1.003)	< 0.001*	0.003*

*Note:* A multivariate logistic regression model was used to assess the independent association of variables that were statistically significant in the univariate analysis.

Abbreviation: IDWG: interdialytic weight gain.

* *p* < 0.05.

Multivariate regression analysis was done, and *p* values were corrected for multiple testing using FDR; the results showed that lower BMI, higher CCI, longer duration on dialysis and lower average weekly treatment time, lower creatinine‐S and higher triglycerides were found to be independent factors associated with higher odds of developing nutritional risk. Factors such as age, gender, history of diabetes, HDF modality, NPCR, equilibrated Kt/V, urea reduction ratio, predialysis urea levels, haemoglobin levels, phosphorus, calcium–phosphorus product and HDL cholesterol although significant in univariate analyses were not identified as independent predictors of nutritional risk in multivariate analysis.

### 3.5 Sarcopenic Parameters Among Nutritional‐Risk Population

Average SARC‐F, SARC‐CalF score and SARC‐CalF (Adj. CC) scores (mean ± SD) among individuals with high nutritional risk were 3.4 ± 3.0, 10.7 ± 5.3 and 12.6 ± 4.1, respectively. Similarly, among individuals with no nutritional risk, the average scores (mean ± SD) of SARC‐F, SARC‐CalF and SARC‐CalF (Adj. CC) were 1.2 ± 1.8, 6.9 ± 5.3 and 9.7 ± 4.1, respectively. All sarcopenic parameters were significantly higher in patients with high nutritional risk (MIS > 5) compared with those with no nutritional risk (MIS ≤ 5) 4 at *p* value < 0.001*.

### 3.6 Sarcopenia Analysis and Associated Risk Factors

In the study cohort, 1978 (53.1%) dialysis patients were found to have sarcopenia based on the SARC‐CalF (Adj. CC) criteria. Univariate binary logistic regression was performed with patients identified with sarcopenia as the dependent variable and factors deemed clinically relevant in occurrence of sarcopenia as independent risk factors. Several demographic and clinical factors were significantly associated with increased odds of development of sarcopenia. These factors include higher age, female gender, higher comorbidity index, history of diabetes, longer period on dialysis, patients undergoing high‐flux HD, use of tunnelled catheters (in comparison with autologous AV fistula) for primary vascular access and lower IDWG. Regarding laboratory parameters, the odds of developing sarcopenia in dialysis patients were higher among individuals with lower NPCR, lower creatinine levels, lower intact PTH values, higher equilibrated Kt/V, higher urea reduction ratio, lower predialysis urea levels, lower phosphorus, lower calcium–phosphorus product and higher triglyceride levels. Multivariate regression analysis was done, and *p* values were corrected for multiple testing using FDR; the results showed that individuals with higher age, higher comorbidity index and on longer months on dialysis were at increased odds of developing sarcopenia. Similarly, lower serum creatinine levels and higher triglycerides emerged as statistically significant independent risk factors that increased the odds of developing sarcopenia among dialysis patients (Table 3).

**TABLE 3:** Regression analysis of factors associated with sarcopenia risk among dialysis patients.

Total no. of patients, *n* (%)	With sarcopenia	Without sarcopenia	Univariate regression analysis		Multivariate regression analysis		
	1978 (53.1)	1744 (46.9)	Unadjusted OR (95% CI)	*p*	Adjusted OR (95% CI)	*p*	*p* (corrected for multiple testing using FDR)
Demographic variables							
Age, mean ± SD	59.1 ± 15.2	47.3 ± 14.2	1.054 (1.049–1.059)	< 0.001*	1.025 (1.017–1.033)	< 0.001*	0.004*
Gender, *n* (%), (female vs. male)			1.753 (1.537–2)	< 0.001*	1.174 (0.987–1.397)	0.07	0.148
Female	981 (61.0)	627 (39.0)					
Male	997 (47.2)	1117 (52.8)					
Clinical variables							
Body mass index (kg/m^2^), mean ± SD	26.7 ± 6.4	26.9 ± 6.4	0.994 (0.984–1.004)	0.205	0.989 (0.977–1.002)	0.092	0.175
Charlson comorbidity index, mean ± SD	5.43 ± 2.2	3.78 ± 1.8	1.489 (1.437–1.544)	< 0.001*	1.264 (1.173–1.363)	< 0.001*	0.004*
H/o diabetes, *n* (%), (yes vs. no)			2.431 (2.13–2.774)	< 0.001*	1.003 (0.797–1.261)	0.982	0.991
No	827 (42.7)	1109 (57.3)					
Yes	1151 (64.4)	635 (35.6)					
Dialysis variables							
HD modality type, *n* (%), (HDF vs. high‐flux haemodialysis)			0.634 (0.554–0.726)	< 0.001*	0.834 (0.706–0.984)	0.032*	0.087
HDF	616 (45.9)	726 (54.1)					
High‐flux haemodialysis	1362 (57.2)	1018 (42.8)					
Months on dialysis, mean ± SD	74 ± 63.5	68 ± 60.2	1.002 (1–1.003)	0.004*	1.004 (1.002–1.005)	< 0.001*	0.004*
Primary vascular access type (at end of period), *n* (%), (autologous as reference category)							
Autologous AV fistula	1575 (52.3)	1435 (47.7)					
AV graft	75 (51.0)	72 (49.0)	0.949 (0.681–1.322)	0.757	0.657 (0.452–0.956)	0.028*	0.087
Tunnelled catheter	328 (58.1)	237 (41.9)	1.261 (1.051–1.512)	0.012*	1.257 (1.003–1.575)	0.047*	0.112
IDWG (weight gain %), mean ± SD	2.15 ± 1.16	2.24 ± 1.14	0.934 (0.883–0.987)	0.016*	1.021 (0.95–1.097)	0.575	0.684
Weekly treatment time (average, last 4 weeks) (min), mean ± SD	721.5 ± 59.7	718.4 ± 66.9	1.001 (1–1.002)	0.143			
Labs, mean ± SD							
Normalised protein catabolic rate (g/kg/day)	0.97 ± 0.23	1.03 ± 0.24	0.347 (0.263–0.458)	< 0.001*	1.013 (0.108–9.462)	0.991	0.991
Creatinine‐S (mg/dL)	8.42 ± 2.32	10.14 ± 2.75	0.763 (0.742–0.785)	< 0.001*	0.864 (0.83–0.9)	< 0.001*	0.004*
Intact PTH (pg/mL)	384.96 ± 363.12	421.84 ± 404.87	1 (1–1)	0.004*	1 (1–1)	0.39	0.57
Equilibrated Kt/V	1.57 ± 0.23	1.54 ± 0.2	1.73 (1.281–2.335)	< 0.001*	1.891 (0.654–5.462)	0.239	0.413
Urea reduction ratio	0.78 ± 0.05	0.77 ± 0.04	13.267 (2.745–64.115)	< 0.001*	0.237 (0.002–36.877)	0.576	0.684
Predialysis urea (mmol/L)	17.2 ± 4.74	18.61 ± 5.07	0.943 (0.931–0.956)	< 0.001*	0.986 (0.889–1.094)	0.796	0.89
Calcium‐S (mg/dL)	9.06 ± 0.55	9.06 ± 0.58	0.993 (0.886–1.113)	0.902			
Phosphorus‐S, inorganic (mg/dL)	4.76 ± 0.93	5 ± 0.97	0.763 (0.711–0.819)	< 0.001*	0.917 (0.712–1.182)	0.504	0.684
Calcium phosphorus product (mg^2^/dL^2^)	43.19 ± 9.09	45.31 ± 9.28	0.975 (0.968–0.982)	< 0.001*	1.012 (0.986–1.038)	0.361	0.57
Haemoglobin‐B (g/dL)	10.84 ± 0.95	10.86 ± 0.93	0.972 (0.907–1.04)	0.409			
Vitamin D3, 25‐hydroxy‐S (nmol/L)	73.02 ± 48.62	70 ± 46	1.001 (1–1.003)	0.053			
HDL cholesterol‐S (mg/dL)	43.32 ± 13.61	43.2 ± 13.24	1.001 (0.996–1.005)	0.779			
Triglycerides (mg/dL)	59.84 ± 88.73	52.12 ± 83.47	1.001 (1–1.002)	0.007*	1.002 (1.001–1.003)	< 0.001*	0.004*

*Note:* A multivariate logistic regression model was used to assess the independent association of variables that were statistically significant in the univariate analysis.

Abbreviation: IDWG: interdialytic weight gain.

* *p* < 0.05.

### 3.7 Comparison of Nutritional Syndromes/Groups

The key groups outlined in Figure 2 were analysed and compared (Table 4) and the findings with statistical significance are highlighted. The various nutritional syndromes were compared with ‘no nutritional or sarcopenic risk’ group as the reference population. A higher percentage of females and individuals with a history of diabetes were found to be affected by the nutritional syndromes. Key groups not previously discussed so far include the patients with SO (*n* = 285) and patients with both malnutrition and sarcopenia (M + S) (*n* = 1175).

**TABLE 4:** Comparison of patients with and without sarcopenia and nutritional risk according to SARC‐CalF (Adj. CC) and MIS criteria.

Total no. of patients, *n* (%)	Without sarcopenia 1744 (46.9)		With sarcopenia 1978 (53.1)			*p*
	No nutritional or sarcopenic risk	Malnutrition without sarcopenia	Malnutrition with sarcopenia	Sarcopenic nonobesity	Sarcopenic obesity	
	1262 (33.9)	482 (13)	1175 (31.6)	518 (13.9)	285 (7.7)	
Demographic variables						
Age, *n* (%)	46.66 ± 13.67	49.1 ± 15.44	61.17 ± 15.31	56.07 ± 15.58	56.11 ± 12	< 0.001
	Ref	0.002*	< 0.001*	< 0.001*	< 0.001*	
Gender, *n* (%)						< 0.001
Female	437 (27.2)	190 (11.8)	612 (38.1)	225 (14.0)	144 (9.0)	
Male	825 (39.0)	292 (13.8)	563 (26.6)	293 (13.9)	141 (6.7)	
Clinical variables						
Body mass index (kg/m^2^), *n* (%)	27.71 ± 6.34	24.88 ± 6.21	25.42 ± 6.38	24.94 ± 3.2	34.91 ± 4.66	< 0.001
	Ref	< 0.001*	< 0.001*	< 0.001*	< 0.001*	
Charlson comorbidity index, mean ± SD	3.65 ± 1.73	4.11 ± 1.94	5.75 ± 2.24	4.86 ± 2.09	5.12 ± 2.02	< 0.001
	Ref	< 0.001*	< 0.001*	< 0.001*	< 0.001*	
H/o diabetes, *n* (%)						< 0.001
No	805 (41.6)	304 (15.7)	465 (24.0)	259 (13.4)	103 (5.3)	
Yes	457 (25.6)	178 (10.0)	710 (39.8)	259 (14.5)	182 (10.2)	
Dialysis variables						
HD modality type, *n* (%)						< 0.001
HDF	517 (38.5)	209 (15.6)	351 (26.2)	151 (11.3)	114 (8.5)	
High‐flux haemodialysis	745 (31.3)	273 (11.5)	824 (34.6)	367 (15.4)	171 (7.2)	
Months on dialysis, mean ± SD	63.72 ± 59.72	79.24 ± 60.25	78.94 ± 65.46	69.1 ± 64.41	61.69 ± 50.01	< 0.001
	Ref	< 0.001*	< 0.001*	0.095	0.616	
Primary vascular access type (at end of period), *n* (%)						0.052
Autologous AV fistula	1046 (34.8)	389 (12.9)	929 (30.9)	416 (13.6)	230 (7.6)	
AV graft	46 (31.3)	26 (17.7)	45 (30.6)	14 (9.5)	16 (10.9)	
Tunnelled catheter	170 (30.1)	67 (11.9)	201 (35.6)	88 (15.6)	39 (6.9)	
EPO dose per week, mean ± SD	4914.46 ± 3988.11	5127.59 ± 4054.57	5482.77 ± 4203.05	5102.32 ± 3837.13	4935.09 ± 4507.64	0.012
	Ref	0.33	0.001*	0.378	0.939	
IDWG (weight gain %), mean ± SD	2.24 ± 1.13	2.25 ± 1.19	2.16 ± 1.19	2.15 ± 1.21	2.11 ± 0.95	0.178
	Ref	0.832	0.099	0.140	0.089	
Weekly treatment time (average, last 4 weeks) (min), mean ± SD	719.95 ± 65.1	714.36 ± 71.48	718.18 ± 63.7	724.27 ± 57.6	729.79 ± 42.76	0.007
	Ref	0.098	0.49	0.189	0.017*	
Karnofsky index, mean ± SD	89.39 ± 11.5	86.8 ± 14	76.6 ± 17.93	83.42 ± 14.68	81.44 ± 15.8	< 0.001
	Ref	0.001*	< 0.001*	< 0.001*	< 0.001*	
Morse Fall Scale score, *n* (%)						
No risk	667 (42.5)	234 (14.9)	373 (23.8)	203 (12.9)	92 (5.9)	< 0.001
Low to moderate risk	569 (29.9)	224 (11.8)	651 (34.2)	284 (14.9)	177 (9.3)	
High risk	25 (10.1)	24 (9.7)	151 (61.1)	31 (12.6)	16 (6.5)	
Not available	1 (0.1)					
Labs, mean ± SD						
Normalised protein catabolic rate (g/kg/day)	1.05 ± 0.24	0.98 ± 0.23	0.95 ± 0.23	0.99 ± 0.24	1 ± 0.22	< 0.001
	Ref	< 0.001*	< 0.001*	< 0.001*	0.002*	
Creatinine‐S (mg/dL)	10.35 ± 2.82	9.61 ± 2.49	7.97 ± 2.2	8.94 ± 2.27	9.32 ± 2.43	< 0.001
	Ref	< 0.001*	< 0.001*	< 0.001*	< 0.001*	
Intact PTH (pg/mL)	416.45 ± 393.43	435.97 ± 433.5	382.35 ± 412.56	383.47 ± 270.35	398.4 ± 284.64	0.042
	Ref	0.342	0.028*	0.099	0.473	
Equilibrated Kt/V (from lab)	1.53 ± 0.19	1.55 ± 0.22	1.59 ± 0.25	1.55 ± 0.22	1.51 ± 0.15	< 0.001
	Ref	0.075	< 0.001*	0.171	0.056	
Urea reduction ratio	0.77 ± 0.04	0.78 ± 0.04	0.78 ± 0.04	0.77 ± 0.05	0.77 ± 0.03	< 0.001
	Ref	0.087	< 0.001*	0.27	0.066	
Predialysis urea (mmol/L)	19.08 ± 5.07	17.38 ± 4.88	16.71 ± 4.72	17.82 ± 4.68	18.15 ± 4.71	< 0.001
	Ref	< 0.001*	< 0.001*	< 0.001*	0.003*	
Calcium‐S (mg/dL)	9.07 ± 0.58	9.05 ± 0.58	9.05 ± 0.57	9.07 ± 0.54	9.08 ± 0.49	0.842
	Ref	0.53	0.431	0.824	0.786	
Phosphorus‐S, inorganic (mg/dL)	5.05 ± 0.99	4.85 ± 0.89	4.7 ± 0.98	4.79 ± 0.86	4.95 ± 0.85	< 0.001
	Ref	< 0.001*	< 0.001*	< 0.001*	0.082	
Calcium phosphorus product (mg^2^/dL^2^)	45.82 ± 9.38	43.98 ± 8.89	42.62 ± 9.54	43.53 ± 8.31	44.92 ± 8.32	< 0.001
	Ref	< 0.001*	< 0.001*	< 0.001*	0.132	
Haemoglobin‐B (g/dL)	10.91 ± 0.9	10.76 ± 1.01	10.82 ± 0.97	10.79 ± 0.96	11 ± 0.8	< 0.001
	Ref	0.003*	0.028*	0.017*	0.135	
Total iron‐binding capacity‐S (μmol/L)	39.87 ± 8.33	35.39 ± 7.79	35.83 ± 8.02	42.13 ± 8.72	40.18 ± 8.79	< 0.001
	Ref	< 0.001*	< 0.001*	< 0.001*	0.575	
Albumin‐S (g/L)	39.38 ± 2.55	38.22 ± 2.67	37.8 ± 2.85	39.13 ± 2.42	38.91 ± 2.29	< 0.001
	Ref	< 0.001*	< 0.001*	0.071	0.006*	
Vitamin D3, 25‐hydroxy‐S (nmol/L)	69.79 ± 46	70.54 ± 46.05	71.43 ± 46.76	79.52 ± 55.48	67.76 ± 41.18	< 0.001
	Ref	0.767	0.393	< 0.001*	0.513	
HDL cholesterol‐S (mg/dL)	42.72 ± 13.18	44.44 ± 13.32	43.85 ± 13.79	43.93 ± 14.01	40.02 ± 11.51	< 0.001
	Ref	0.017*	0.038*	0.083	0.002*	
Triglycerides (mg/dL)	52.73 ± 86.62	50.52 ± 74.66	63.03 ± 82.28	46.98 ± 85.23	70.05 ± 114.68	< 0.001
	Ref	0.632	0.003*	0.201	0.002*	

* *p* < 0.05.

The group with combined malnutrition and sarcopenia (M + S) (31.6% patients) has a significantly higher age, higher number of females, lower BMI, higher CCI, higher percentage of diabetes, lower Karnofsky performance status score, increase fall risk based on Morse fall score, longer months on dialysis, higher weekly EPO dosage, lower levels of creatinine, lower NPCR, lower Intact PTH, higher predialysis urea, higher equilibrated Kt/V, higher urea reduction ratio, lower predialysis urea, lower phosphorous, lower calcium phosphorus product, lower TIBC, lower haemoglobin‐B, higher HDL and higher triglycerides compared with the cohort with no nutritional or sarcopenia risk. Furthermore, in this combined (M + S) group, 61.1% were classified as being at high risk of falls (Morse scale), all while having an average borderline overweight BMI of 25.42 ± 6.38 kg/m^2^.

## 4 Discussion

This is the first large‐scale, multicentre study to characterise malnutrition and sarcopenia risk in a Saudi ESKD HD population. A central and novel finding is that malnutrition and sarcopenia risk are highly prevalent even in this relatively young cohort. With regard to sarcopenia, this finding is clinically meaningful because the revised EWGSOP2 consensus explicitly distinguishes between primary (age‐related) sarcopenia and secondary sarcopenia, the latter driven by systemic disease, chronic inflammation, inactivity or inadequate nutrition [16]. CKD and maintenance HD exhibit all four of these catabolic drivers: uremic toxicity, persistent low‐grade inflammation, sedentary behaviour and PEW, making the sarcopenia construct both applicable and clinically relevant in younger dialysis patients. To compare, a recent systematic review of dialysis populations investigating sarcopenia prevalence found the average age of patients was 62.3 years across 41 studies (*n* = 7576) [34]. In our cohort, sarcopenia risk was identified in 53.1% of patients, with mean ages of 57.7 ± 16.3 years in those with malnutrition and 59.1 ± 15.2 years in those with sarcopenia.

Sarcopenia data from Middle Eastern HD cohorts remain scarce. The only regional comparator is a 2019 Egyptian study (*n* = 37) reporting 35.1% sarcopenia prevalence by EWGSOP criteria, though the small sample limits direct comparison [35]. Globally, two recent systematic reviews of dialysis populations have reported pooled prevalences of 25.6% (7576 patients across 41 studies) [34] and 25.9%–34.6% (6162 patients across 30 studies) [19]. Both reviews linked sarcopenia to increased mortality, and Wathanavasin et al. further reported independent associations with cardiovascular events and with both low MM and low muscle strength [34]. In the most recent systematic review of sarcopenia in CKD in global studies by Duarte et al., they found that muscle weakness was highest in dialysis patients (50%), compared with nondialysis (19.6%) patients [18].

This study also highlights a potentially clinically important blind spot: 21.6% of patients were flagged as being at sarcopenia risk without meeting nutritional‐risk criteria on MIS. This is relevant in HD populations where higher adiposity or stable BMI may mask muscle wasting, and thus, the conventional reliance on BMI and weight‐based screening alone is inadequate. SO was identified in 7.7% of our cohort, consistent with the 10.8% pooled prevalence reported in a recent systematic review of CKD populations [18]. Together, these findings reinforce the need for routine, multitool screening that captures both nutritional and skeletal muscle dimensions (e.g., mass and function) in HD care.

The utilisation of the SARC‐Calf tool is novel in this Saudi HD population group and the SARC‐CalF (Adj. CC)‐based sarcopenia risk prevalence is higher than global average estimates. However, SARC‐F and SARC‐CalF with nonadjusted CC‐based prevalence were more in line with global averages (Figure 4). Much of the variation noted centres on the use of the CC measurement (for completing a SARC‐CalF score) and after adjustment for BMI and presence of oedema. It should be noted that prevalence of low MM after adjustment was very high (74.7% had severely low levels), and whether this is wholly accurate is unknown at present. Questions surround the appropriateness of the use of the suggested cut‐off points before adjustment and/or use of specific adjustment factors for the Saudi population group. This requires future investigation and validation studies comparing accuracy to validated MM assessment techniques (e.g., comparison with MRI) in this population group. Duarte et al. found that the SARC‐CalF had much higher sensitivity in detecting probable sarcopenia, than the SARC‐F comparing to hand grip strength measurements and five‐times sit‐to‐stand testing, which was interesting, although a small sample size was studied [24].

The malnutrition risk prevalence in this study (44.5% by MIS) is consistent with the broader regional picture, though tool heterogeneity may account for some variation across reports. Earlier KSA single‐ or two‐centre studies using SGA‐based tools have reported malnutrition prevalences of 48.7%–55.0% in HD patients (*n* = 211–269) [15]. Smaller studies from neighbouring Middle Eastern countries (*n* = 55–185) have reported broadly comparable estimates. In Palestine, 45.4% of 153 HD patients were at high malnutrition risk by the Renal iNUT tool [36], while a more recent West Bank cohort (*n* = 188, mean age 57.8 ± 14 years) using MIS, the most directly comparable methodology to ours, reported a lower prevalence of 28.2% [37] The study MIS‐based estimate sits closer to SGA‐based KSA studies than to the West Bank MIS cohort, likely reflecting differences in population case mix, dialysis vintage and comorbidity burden rather than tool choice alone.

In this cohort, 31.6% of patients had both malnutrition and sarcopenia (Figure 3 and Table 4), the largest single subgroup after those with neither condition. Compared with the no‐risk reference group, the M + S group was significantly older and had a higher CCI and higher diabetes prevalence (39.8%); longer dialysis vintage; lower Karnofsky score; higher falls risk; and lower albumin, TIBC and NPCR (all *p* < 0.001, Table 4). This clinical profile, combining reduced functional capacity, inflammatory biomarkers and reduced dietary protein intake, closely mirrors the Evans et al. phenotypic criteria for cachexia and the multifactorial framework of ‘PEW progressing to cachexia’ [10, 38], and previous HD studies have shown that combined malnutrition and sarcopenia independently increase mortality risk [39]. These patients are likely to benefit most from targeted, multidisciplinary/multimodal interventions combining nutritional support, physical activity (e.g., resistance exercise) and management of inflammation and comorbidity, which indeed has been the focus in recent clinical trials for cachexia across chronic conditions, such as CKD [40], though the relative contribution and sequencing of these components in HD populations remains an open question. Importantly, the M + S phenotype would be systematically missed by BMI‐ and weight‐based screening alone, reinforcing the case for routine multitool assessment in HD care.

This study identified several common factors that were associated with both malnutrition and sarcopenia, while also demonstrating that certain factors were uniquely associated with either malnutrition or sarcopenia alone. Specifically, while lower BMI is independently associated with malnutrition risk, sarcopenia risk is BMI‐independent. Additionally, it can be inferred that promoting AV fistula use especially for younger patient population and adherence to prescribed treatment time may be of clinical value, not only for dialysis adequacy (Kt/V) but also to potentially sarcopenia and functional decline. In addition, the use of some blood biomarkers specifically serum creatinine as a low‐cost proxy measure for MM may have potential added value in malnutrition and sarcopenia diagnosis and warrant further exploration.

Although gender did not emerge as an independent predictor in regression models, female patients in our study had significantly higher average scores on CC, SARC‐F and SARC‐CalF (Adj. CC), compared with males (Table 1). Similarly, diabetes, NPCR, equilibrated Kt/V, urea reduction ratio, predialysis urea, phosphorus, calcium–phosphorus product and PTH were not significantly associated with both conditions in the multivariate regression despite their significant association in univariate analysis (Tables 2 and 3). This suggests that their effect is likely mediated through other variables including comorbidity burden, inflammation, HD modality or vascular access. Note that 25‐OH vitamin D3 and haemoglobin levels are managed through use of supplementation and medication; standardised clinical protocols, on the other hand, did not emerge as a top‐level predictor for sarcopenia (Table 4).

This study is not without limitations. First, the use of screening tools such as MIS, SARC‐F and SARC‐CalF with adjusted CC, while practical, does not substitute for gold standard assessments for body composition or functional performance. Additionally, established cut‐off points particularly for CC may lack accuracy for the younger and more overweight KSA population and call for local validation. Furthermore, we acknowledge that multiple clinical dietitians took measurements across multiple clinics; however, standardised training and measurement protocols were established to reduce possible variability and error in measurements. Finally, we acknowledge the limitation of the lack of assessment of physical activity in our study population. Physical activity/inactivity plays a crucial role in the development of sarcopenia, and the absence of individual‐level physical activity data may lead to residual confounding, and the observed associations should be interpreted accordingly.

## 5 Conclusion

This is the first large‐scale, multicentre study to assess malnutrition and sarcopenia risk in a Saudi and Middle Eastern HD population using standardised, guideline‐based screening tools. Conducted as part of the Project Muscle initiative, it addresses a significant regional evidence gap by characterising the prevalence and co‐occurrence of malnutrition, sarcopenia, SO and PEW/cachexia features in a relatively young dialysis cohort, with all prevalence estimates substantially elevated. These findings support routine, multitool nutritional and muscle screening as the critical first step towards individualised, patient‐centred intervention in HD care. More detailed analyses using in‐depth anthropometry, body composition and biomarker data are underway as part of Project Muscle to build on the foundation established here.

## Ethics Statement

All authors certify that this manuscript complies with the ethical guidelines for authorship and publishing in the *Journal of Cachexia, Sarcopenia and Muscle*. Full research ethics were sought and confirmed by the IRB: King Fahad Medical City Institutional Review Board (21‐519E and 24‐715E) prior to the study commencing.

## Conflicts of Interest

The study was supported by the private company Diaverum, and hence, all team members are paid employees by the company, apart from J.R., and A.S. was a paid external consultant.
